# Nickel substituted polyoxometalates in layered double hydroxides as metal-based nanomaterial of POM–LDH for green catalysis effects

**DOI:** 10.1038/s41598-023-31356-7

**Published:** 2023-03-13

**Authors:** Azra Ghiasi Moaser, Ahmad Gholami Afkham, Roushan Khoshnavazi, Sadegh Rostamnia

**Affiliations:** 1 Organic and Nano Group (ONG), Department of Chemistry, Iran University of Science and Technology (IUST), P.O. Box 16846-13114, Tehran, Iran; 2 Department of Chemistry, University of Kurdistan, P.O. Box 66135-416, Sanandaj, Iran

**Keywords:** Materials science, Chemistry, Catalysis

## Abstract

Three nickel substituted Keggin-type polyoxometalates, α-[SiW_9_O_37_{Ni(H_2_O)}_3_]^−10^ (denoted as SiW_9_Ni_3_), was intercalated into Zn_3_Al based Layered Double Hydroxide (Zn_3_Al-LDH) by the selective ion-exchange technique. The as-synthesized nanocomposite, SiW_9_Ni_3_@Zn_3_Al, was used as heterogeneous nanoreactor to promote the synthesis of drug-like aminoimidazopyridine small molecule skeletons via the well-known Ugi-type Groebke–Blackburn–Bienaymé reaction (GBB 3-CRs) in the absence of any acid/additive and under mild and solvent-free conditions. A synergistic catalytic effect between SiW_9_Ni_3_ polyoxometalate and Zn_3_Al-LDH precursors is evidenced by a higher catalytic property of the SiW_9_Ni_3_@Zn_3_Al composite compared to the individual constituents separately. Lewis/Bronsted acidity of the SiW_9_Ni_3_ polyoxometalate and Zn_3_Al-LDH precursors appear to be essential for the catalytic performance of the composite. Furthermore, the catalytic performance of SiW_9_Ni_3_@Zn_3_Al was also tested in GBB 3-CRs synthesis of amino imidazothiazole under mild and solvent-free conditions.

## Introduction

The ‘greening’ of the global chemical processes has become an important challenge in chemical industry^1^. Green chemistry provides “Green” paths for reactions not only via reducing of byproducts, produced waste, energy costs and materials consumption but also by well advised in the selection of nonhazardous solvents and green catalysts^2^. Furthermore, in development of efficient green synthesis procedures, Solvent-free (S-F) approach has become a major focus of researchers due to advantages over the classical method of synthesis. The S-F procedure decreases the use of organic toxic solvents and Volatile Organic Compounds (VOCs) and minimizes the formation of other wastes^3,4^. On the other hand, development of various approaches for heterogenizing of homogeneous catalysts, which minimizes consumption of materials like solvents, energy and time, can result in significant economic and environmental benefits. Therefore, from both environmental and economic viewpoints, organic reactions under solvent-free and recoverable catalyst conditions have gained considerable interest in recent years^5^.

Multicomponent reactions (MCRs) with combinatorial methods have been used as a convenient approach toward the synthesis of various classes of compounds^6–9^. The isocyanide-based multicomponent reactions (IMCRs), such as the versatile well-known Ugi, Passerini and Oakes-Yavari-Nair (OYN) reactions^10^, is one of the pivotal reactions in this area^11^. Due to antifungal and antibacterial activities of some aminoimidazo[1,2-a]pyridines, these small drug-like molecules are important class of pharmaceutical compounds^11^. To date, a number of Lewis and Bronsted acids such as acetic acid, TsOH, Cell-SO_3_H, RuCl_3_, MOFs^12^, MgCl_2_, SnCl_2_, ZrCl_4_, and ZnCl_2_ have been applied for the synthesis of aminoimidazopyridines via GBB 3-CRs^13^. Given that some of these catalytic systems suffer from low yields, harsh reactions conditions, long reaction times, tiresome work-up which lead to the generation of large amounts of toxic waste and co-occurrence of several side reactions^14–22^. Afterwards, some of them are impossible to use because of the economy/environmental considerations. Correspondingly, there is enough space for the development of new synthetic methods as an attractive goal.

Polyoxometalates (POMs) are a large group of inorganic anionic clusters, which mostly composed of oxo-bridged early transition metals (TMs) such as tungsten, molybdenum, vanadium, etc., in their highest oxidation states^23^. Owing to their structural versatilities and tunable chemical and physical properties such as redox behavior, Lewis/Bronsted acidity, molecular structure diversities and high negative charges, they have been applied in a wide range of fields including catalysis, medicine, materials and environment^24–27^. To date, a wide variety of POMs have generally been applied as acid and oxidation catalysts, especially Bronsted acids. Albeit, their use as Lewis acid catalysts is limited due to occupation of d orbitals of high-valent metal centers with the surface oxo ligands^28,29^. In this case, to develop the POMs as catalysts, the physical and chemical properties of them can be adjusted by incorporation of transition-metals into their framework, which can create catalytically active sites in the structure of POMs^30,31^. However, a problem in POMs applications lies in the necessity of converting soluble POMs to solid materials due to their relatively low surface area (< 10 m^2^ g^−1^) and high solubility in polar solvents^32^. Thus, heterogenizing of POMs makes them encouraging candidates as nanocatalysts for various kinds of chemical reactions and green chemistry^33,34^. On the basis of previous reports, intercalation of POMs into Layered Double Hydroxides (LDH) is a way to develop the heterogenized POMs-based catalysts heterogeneous catalysts with unique properties. LDHs with general formula [M^2+^_1−x_M^3+^_x_(OH)_2_]^x+^(A^n−^)_x/n_·yH_2_O, are a large class of positively charged brucite-like layers with building blocks of divalent and trivalent metal cations as well as exchangeable anions such as Cl^−^, CO_3_^2−^, NO^3−^ between the layers.

**Figure 1:**
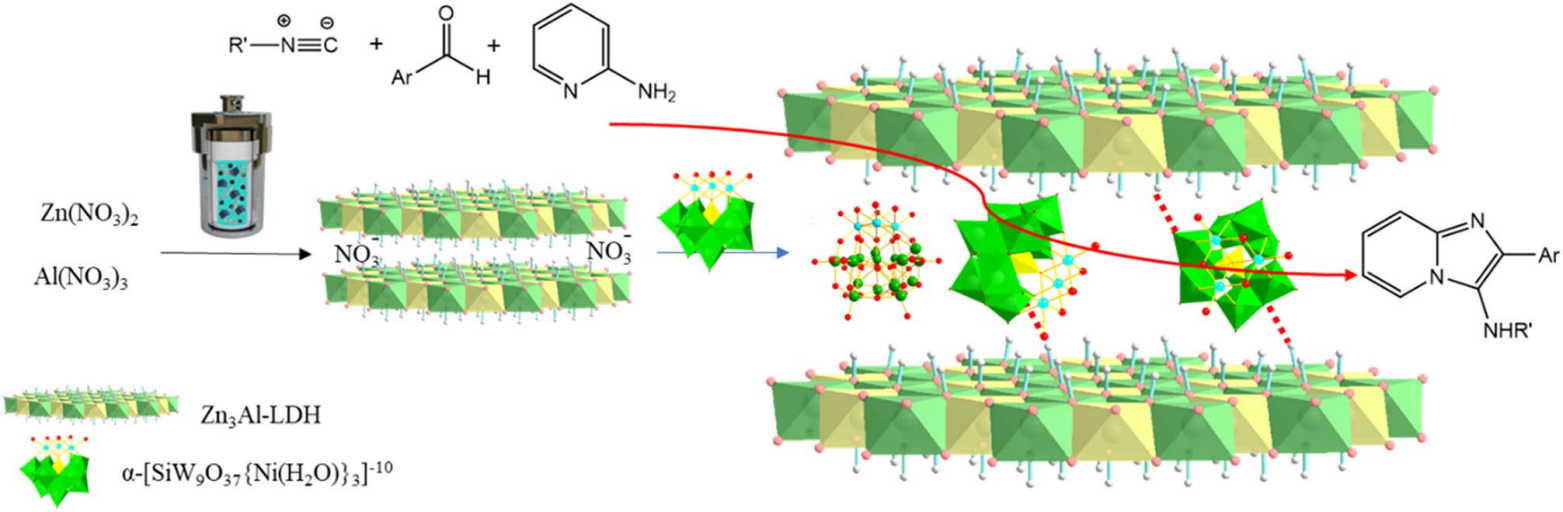
Illustration of the procedure for the SiW_9_Ni_3_@Zn_3_Al nanocomposite and the synthesis route of amino imidazothiazole over the POM–LDH catalyst.

In this work, the nanoreactor of a Zn_3_Al–NO_3_ LDH pillared with the Keggin-type three-nickel-substituted of α-[SiW_9_O_37_{Ni(H_2_O)}_3_]^−10^ anions as a atomically thin-type materials were synthesized and confirmed structurally with various techniques including TGA, FT-IR, SEM, X-ray diffraction (XRD), energy dispersive X-ray (EDX), transmission electron microscopy (TEM), Brunauer–Emmett–Teller (BET) and zeta potential. Here we focus on catalytic application of nickel substituted polyoxometalate intercalated Zn_3_Al–NO_3_ layered double hydroxide as a heterogeneous catalyst for the acid-catalyzed synthesis of small molecule of aminoimidazopyridine via the GBB 3-CRs under mild and S-F conditions without the necessity of any Bronsted acid or additives (Fig. 1).

## Experimental

### Materials and apparatus

All the chemicals were purchased from commercial companies and used without further purification. Powder X-ray diffraction (XRD) patterns were recorded on a Philips XʼPert MPD diffractometer equipped with Cu Kα radiation (λ = 1.54056 Å) and operated at 40 kV 30 mA. FT-IR spectra were recorded on a Bruker model vector 22 Fourier transform spectrophotometer, using KBr Pellet. Surface areas and pore size distributions were investigated using nitrogen physisorption at 77 K on a Micromeritics Tristar II Plus surface area analyzer. The SEM images and corresponding energy dispersive X-ray (EDX) analytical data were determined by using FESEM-TESCAN MIRA3 scanning electron microscope equipped with an EDX detector. TEM was carried out with a Zeiss-EM10C microscope operating at 80 kV. Thermogravimetric analyses (TGA) were performed by a STA PT-1000 LINSEIS apparatus.

### Preparation of SiW_9_Ni_3_@Zn_3_Al nanocomposite

The preparation of SiW_9_Ni_3_@Zn_3_Al nanocomposite was performed through a three-step procedure: (1) synthesis of α-[SiW_9_O_37_{Ni(H_2_O)}_3_]^−10^, (2) hydrothermal synthesis of the Zn_3_Al–NO_3_ layered double hydroxide and finally (3) intercalation of the [SiW_9_O_37_{Ni(H_2_O)}_3_]^−10^ anions into the Zn_3_Al–NO_3_ via anion exchange process under N_2_ atmosphere. Decarbonated-deionized water is used in experiments. It is prepared by boiling and bubbling nitrogen gas into the deionized water to remove the dissolved CO_2_.

### Synthesis of α-Na_10_[SiW_9_O_34_]·18H_2_O (SiW_9_)

Firstly, 91 g of Sodium tungstate was dissolved in 100 mL of water. After clarifying, 5.5 g of sodium silicate was magnetically dissolved in the above solution. Then 65 mL of HCl acid (6 M) was added to the stirring solution. Next, the mixture was boiled to concentrate it to half of its volume. After cooling down, the solution was filtered, then 20 g of anhydrous sodium carbonate was added to the filtrate. Then, the solution was magnetically stirred for 20 min. Finally, the sodium salt of the a-9-tungstosilicate precipitated. The FT‐IR spectrum matched the literature data^35^.

### Synthesis of α-[SiW_9_O_37_{Ni(H_2_O)}_3_]^−10^ (SiW_9_Ni_3_)

Firstly, 3.4 g (12 mmol) NiSO_4_·7H_2_O was dissolved in 150 mL of sodium acetate (0.5 M). In the next step, 11.2 g (4 mmol) SiW_9_ was added to the solution at 70 ºC. After cooling to room temperature, a solution of 4.2 g KCl in 12 mL distilled water was added to yield an iridescent green product. The obtained precipitate was recrystallized from hot water. The FT-IR spectrum matched the literature data^36^.

### Synthesis of Zn_3_Al–NO_3_ (Zn_3_Al-LDH)

In a typical experiment, a solution of 3.8 g Al(NO_3_)_3_·9H_2_O (0.01 mol) and 7.8 g Zn(NO_3_)_2_·4H_2_O (0.03 mol) in 100 ml decarbonated H_2_O was mixed with a solution of 3.2 g NaOH (0.08 mol) in 100 ml of decarbonated H_2_O. In two minutes, the obtained slurry was transferred to autoclave, aged for 12 h at 100 ºC. It was washed with decarbonated H_2_O and ethanol for several times upon cooling to room temperature^37^.

### Preparation of SiW_9_Ni_3_@Zn_3_Al

In a typical procedure, a solution of 3.2 g SiW_9_Ni_3_ (1.14 mmol) in a 40 mL of decarbonated water was taken dropwise to the slurry of Zn_3_Al–NO_3_ under N_2_ atmosphere while vigorously stirring. Then the resulted green slurry was stirred for 5 h at 60 °C. Finally, the green precipitate of SiW_9_Ni_3_@Zn_3_Al was filtered, washed with decarbonated water and ethanol for several times to remove the unreacted reagents and dried overnight at 60 °C under a vacuum.

### General procedure for S-F preparation of the aminoimidazoles

In a typical procedure, to a mixture of aldehyde (1 mmol) and 2-aminopyridines (1 mmol) the SiW_9_Ni_3_@Zn_3_Al catalyst (1 mol%) was added under S-F condition at room temperature. The resulting solution was then stirred well for 5 min. Afterwards, 1.2 mmol of alkyl isocyanide was taken to the reaction media and stirred well at 35 °C for proper time. Progress of the reaction was checked by TLC. At the end of the reaction, after cooling the reaction mixture, CH_2_Cl_2_ (4 mL) was added. The catalyst was readily recovered (after the adding of CH_2_Cl_2_) from the reaction media using centrifuge (3000 rpm for 10 min) separation. Afterwards, it was washed with diethyl ether and dichloromethane solvents and dried under vacuum to reuse in the next run (ESI).

## Results and discussion

**Figure 2:**
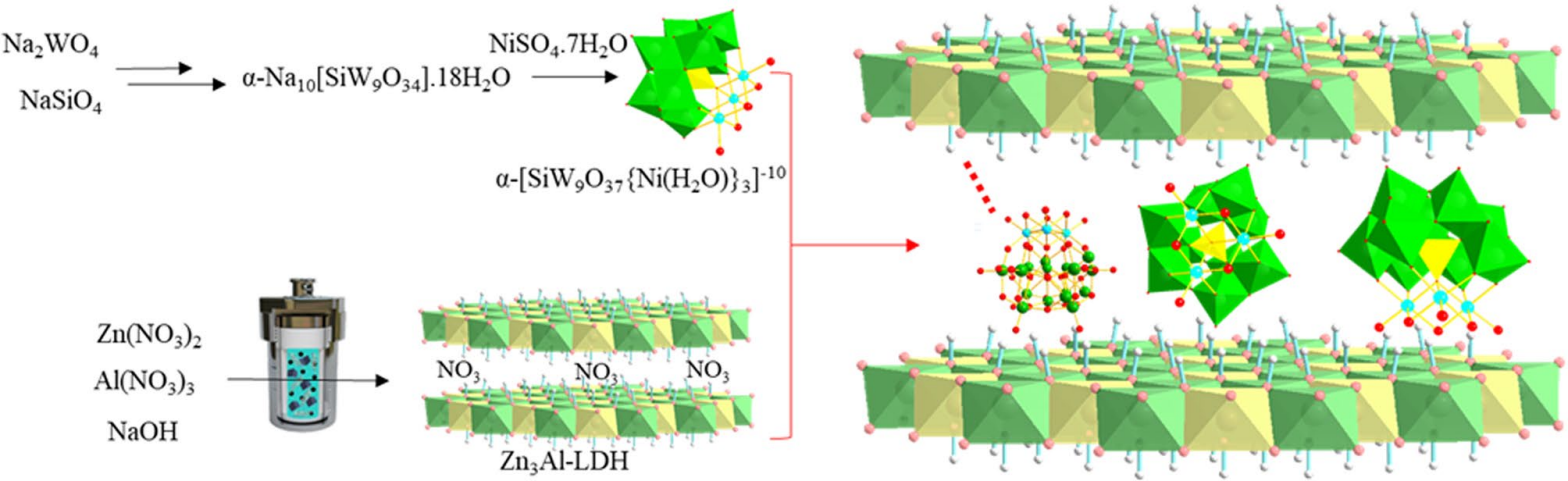
Schematic illustration of the preparation route for the intercalation of SiW_9_Ni_3_ anions into Zn_3_Al-LDH.

Zn_3_Al–NO_3_ LDH was synthesized successfully via hydrothermally treating of an aqueous solution including Zn(NO_3_)_2_·4H_2_O, Al(NO_3_)_3_·9H_2_O and NaOH. Intercalating POM anions of α-[SiW_9_O_37_{Ni(H_2_O)}_3_]^10−^ into the Zn_3_Al–NO_3_ interlayers under N_2_ atmosphere leads to the construction of novel intercalated assembly of SiW_9_Ni_3_@Zn_3_Al nanocomposite (Fig. 2).

**Figure 3:**
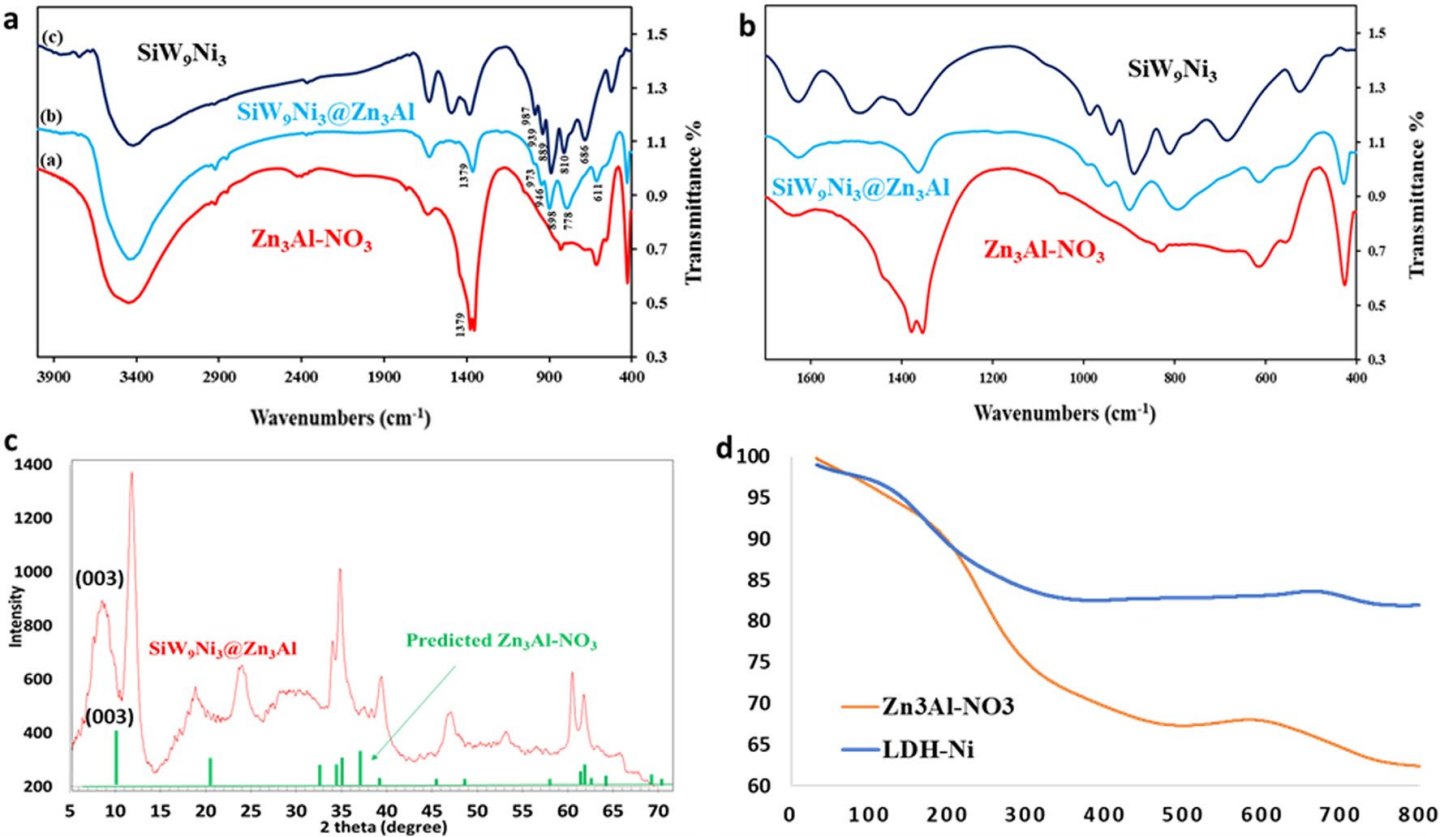
(**a**, **b**) FT-IR spectra of Zn_3_Al–NO_3_, SiW_9_Ni_3_@Zn_3_Al and SiW_9_Ni_3_; (**c**) XRD pattern of Zn_3_Al–NO_3_ and SiW_9_Ni_3_@Zn_3_Al; (**d**) TGA curves of Zn_3_Al–NO_3_ and SiW_9_Ni_3_@Zn_3_Al.

Successful intercalation of SiW_9_Ni_3_ anions into the Zn_3_Al–NO_3_ is proved by comparison of FT-IR spectra of the Zn_3_Al–NO_3_, SiW_9_Ni_3_ and SiW_9_Ni_3_@Zn_3_Al nanocomposite (Fig. 3a,b). The FT-IR spectrum of Zn_3_Al–NO_3_ precursor shows a sharp peak at *ca.* 1379 cm^−1^ which is related to the ν_3_ stretching vibration of nitrate in the interlayer galleries^38^. The intensity of the corresponding band in the spectrum of SiW_9_Ni_3_@Zn_3_Al extremely decreases which indicates the large amount of nitrate anions were exchanged with the guest anions. The FT-IR spectrum of SiW_9_Ni_3_ indicates characteristic peaks at 987, 939, 889 and 810 cm^−1^ attributing to the stretching vibrations of Si–O, W–O_d_, W–O_b_ and W–O_c_, in which d, b, and c represents terminal, corner sharing, and edge sharing oxygen, respectively^36^. These stretching peaks can be clearly observed in the FT-IR spectrum of SiW_9_Ni_3_@Zn_3_Al. The slight shift of the corresponding bands of SiW_9_Ni_3_ in SiW_9_Ni_3_@Zn_3_Al to 973, 946, 898 and 778 respectively can be referred to the hydrogen bonding interactions between LDH layers and POMs anions^38,39^. All these data demonstrate the successful intercalation of guest anions SiW_9_Ni_3_ into the LDH host layers. The XRD patterns of Zn_3_Al–NO_3_ and its intercalated product of SiW_9_Ni_3_@Zn_3_Al are shown in Fig. 3c. For Zn_3_Al–NO_3_, two sharp basal reflections at 2θ = 10° and 2θ = 20° are indexed to (003) and (006) planes respectively^40^. According to the XRD spectroscopy of SiW_9_Ni_3_@Zn_3_Al, when the small nitrate anions are exchanged by large SiW_9_Ni_3_ anions, the space between the layers of lamellar expand and the basal (003) and (006) reflections of SiW_9_Ni_3_@Zn_3_Al shifts to lower position. For SiW_9_Ni_3_@Zn_3_Al composite, the obvious shift of the characteristic diffraction peak (003) to the left compared to the Zn_3_Al–NO_3_ confirms the successful intercalation of POMs. The gallery height value of around 0.98 nm is obtained by subtracting the thickness of the Zn_3_Al–NO_3_ layer (0.48 nm) from the value of d(003) spacing of the SiW_9_Ni_3_@Zn_3_Al, which is in accordance with the diameter of Keggin-type POMs^41^. Furthermore, the diffraction peaks in the XRD pattern of the SiW_9_Ni_3_@Zn_3_Al are obviously broadened due to overlapping of them with the strong characteristics of the polyoxometalates^38^. Besides, all the diffraction peaks related to pristine ZnAl-NO_3_ remain unchanged after the intercalation of SiW_9_Ni_3_ anions, indicating that the crystal structure of layered double hydroxide is retained (Fig. 3c).

The thermal properties of Zn_3_Al–NO_3_ and SiW_9_Ni_3_@Zn_3_Al composite were investigated with TGA thermogram. As can be observed in Fig. 3d, the TGA curves of Zn_3_Al–NO_3_ and SiW_9_Ni_3_@Zn_3_Al, have three weight loss levels. The first lost weight of 7.27% and 1.12% for Zn_3_Al–NO_3_ and SiW_9_Ni_3_@Zn_3_Al is occurred between 50 and 150 °C respectively. This is ascribed to the evaporation of surface moisture and the structurally bonded intercalated water molecules. The second and the largest weight loss of 25% and 15.3% for Zn_3_Al–NO_3_ and SiW_9_Ni_3_@Zn_3_Al at 150 to 500 °C is related to two processes of dehydroxylation and removal of interlayer anions. The second and the largest weight loss of 25% and 15.3% for Zn_3_Al–NO_3_ and SiW_9_Ni_3_@Zn_3_Al at 150 to 500 °C is related to the destruction of the layered structure. The last stage of weight loss for Zn_3_Al–NO_3_ and SiW_9_Ni_3_@Zn_3_Al can be ascribed to the formation of a spinel phase due to the decay of the mixed metal oxide and decomposition of the POM anions on Zn_3_Al LDH during the temperature range of 500–800^42–44^. Furthermore, SiW_9_Ni_3_@Zn_3_Al reveals its superior thermal resistance compared to the Zn_3_Al–NO_3_ at higher temperatures, by increasing residues from 64% of Zn_3_Al LDH to 84% of SiW_9_Ni_3_@Zn_3_Al (Fig. 3d).

**Figure 4:**
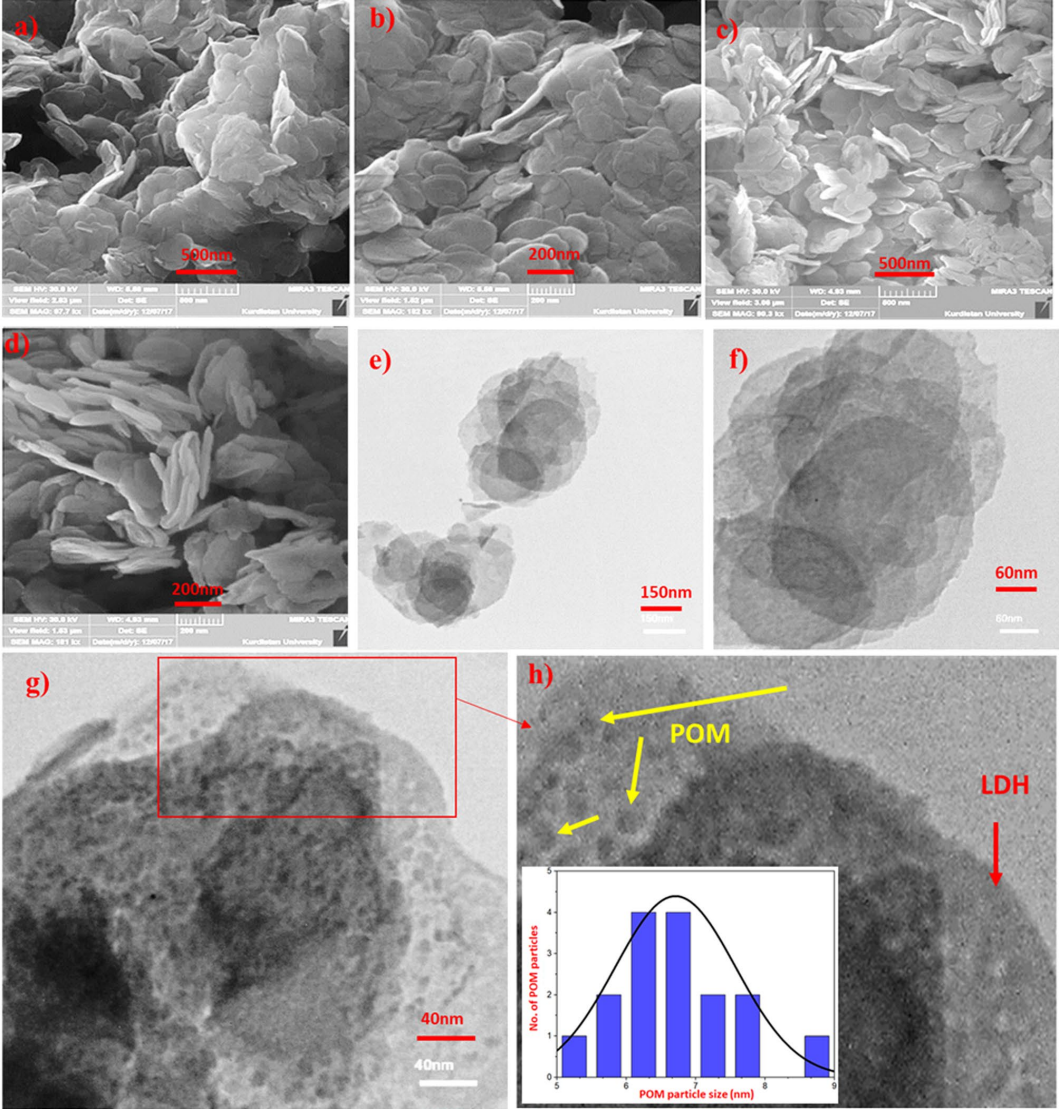
FE-SEM images of (**a**, **b**) Zn_3_Al–NO_3_; (**c**, **d**) SiW_9_Ni_3_@Zn_3_Al; TEM images of (**e**–**h**) SiW_9_Ni_3_@Zn_3_Al. POM particle size histogram for SiW_9_Ni_3_@Zn_3_Al (**h**, inset).

As represented in Fig. 4, the morphological characteristics of Zn_3_Al–NO_3_ and SiW_9_Ni_3_@Zn_3_Al were investigated by SEM and TEM analyses. The pure Zn_3_Al–NO_3_ consists of the irregular hexagonal stacks and plates of LDHs crystallites with the thickness of about 26 nm (Fig. 4a,b). As shown in Fig. 4c,d, after intercalation of SiW_9_Ni_3_ anions, the lamellar structure of Zn_3_Al–NO_3_ plates has not significantly changed, however an obvious separation between the layers can be attributed the effectively hosting of the large polyoxometalates anions. Furthermore, TEM analysis of the SiW_9_Ni_3_@Zn_3_Al represent the Pseudo-hexagonal LDHs lamellae with irregular edges, confirming the results reported from the SEM analysis (Fig. 4e,f). The average size of the Nano sheets is about 300 nm. Moreover, the homogenous distributed dark small dots (yellow arrows) are ascribed to the intercalated SiW_9_Ni_3_ anions (Fig. 4h). The LDH platelets are shown by red arrow (Fig. 4h). The size distribution histogram for POM particles (Fig. 4h, inset) shows a mean diameter of 6.5–7 nm, confirming that POM preserves its monodispersity after immobilization on the LDH support.

**Figure 5:**
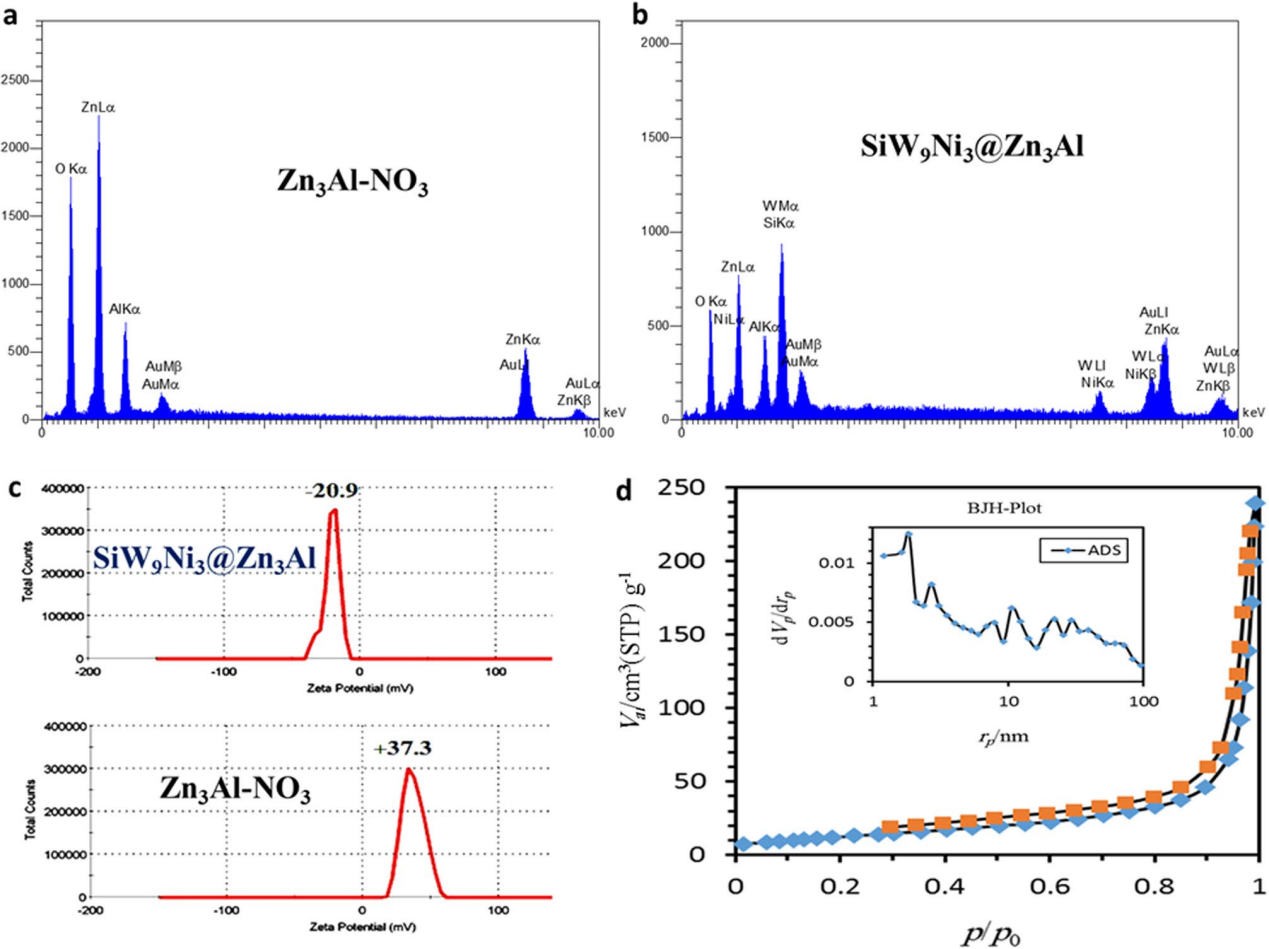
EDX patterns of (**a**) Zn_3_Al–NO_3_ and (**b**) SiW_9_Ni_3_@Zn_3_Al; zeta-potentials of (**c**) Zn_3_Al–NO_3_ and SiW_9_Ni_3_@Zn_3_Al; (**d**) Adsorption–desorption isotherm of SiW_9_Ni_3_@Zn_3_Al.

The EDX results of Zn_3_Al–NO_3_ and Zn_3_Al–SiW_9_Ni_3_ revealed the presence of all the elements of the Zn_3_Al LDH and SiW_9_Ni_3_ anions (Zn, Al, O, W and Ni) in the samples which support this assumption that the SiW_9_Ni_3_ anions have been exchanged successfully with the interlayer NO_3_^−^ anions of Zn_3_Al LDH (Fig. 5a,b). Zeta potential as a standard characterization technique, was used to evaluate and SiW_9_Ni_3_@Zn_3_Al surface charge. According to current study, the interlayer nitrate anions are likely to be exchanged with SiW_9_Ni_3_ anions with high negative charge, leading to increase the surface charge of the nanocomposite. It can be seen in Fig. 5c the zeta potential of the Zn_3_Al–NO_3_ shifted from a positive value (37.3) to a negative value (− 20.9) in SiW_9_Ni_3_@Zn_3_Al nanocomposite. As a result, the increase of the charge density in SiW_9_Ni_3_@Zn_3_Al nanocomposite can be ascribed to the successful intercalation of POM anions into Zn_3_Al-LDH (Fig. 5c). As shown in Fig. 5d the adsorption isotherm of the SiW_9_Ni_3_@Zn_3_Al illustrate a type IV isotherm at lower pressure (P/P0 < 0.1) with H_3_ type hysteresis loops. According to the Brunauer, Deming, Deming and Teller (BDDT) classification the adsorption isotherm confirm the presence of^39,45,46^. Furthemore, BET data revealed that the specific surface area of SiW_9_Ni_3_@Zn_3_Al (46 m^2^/g) is significantly increased in comparison with the reported data of Zn_3_Al–NO_3_ (9 m^2^/g) in other works^40^. This finding probably caused by the interlayer opening due to the existence of POM anion.

After careful characterization of the composite, the catalytic activity of SiW_9_Ni_3_@Zn_3_Al was investigated for the Ugi-like three-component synthesis of imidazopyridines as small biologically interest molecules^11,47,48^. In this study, the reaction between 1 mmol of 2-aminopyridine, 1 mmol of benzaldehyde, and 1.2 mmol of cyclohexyl isocyanide was selected as benchmark model reaction (Table 1S). Due to the important role of solvents in this reaction, the influence of the various solvents as well as solvent-free (S-F) condition were evaluated. Solvents such as toluene, H_2_O, EtOH, MeOH and CH_2_Cl_2_ were used with different times and reaction temperature in the model reaction (Table 1S, entries 1–8). However, the obtained data in Table 1S explains high yield of 3-aminoimidazo[1,2-a]pyridine (94%) synthesized under S-F condition, in 1 h and 35 °C (Table 1S, entry 16). Therefore, the influence of the catalyst dosage, temperature and reaction time was investigated to define the optimum conditions for the three-component synthesis of imidazopyridines, in S-F condition. The results indicate that under the constant amount of catalyst and reaction time, an increase of temperature from 25 to 35 °C resulted in the sharp increase of the yield of the reaction in S-F condition (Table 1S, entries 8, 9). Furthermore, experimental data indicate that increase in temperature results in higher yields in the other solvents as well [50 °C in water (Table 1S, entries 1, 2) and 30 °C in ethanol (Table 1S, entries 4, 5)]. Additionally, the results were clearly demonstrated that by increasing the catalyst dosage in a constant temperature, the yield of the product increased (entries 13 and 16). Moreover, the yield of the product raised by increasing the reaction time from 0.5 to 1 h (ESI).

**Table 1:** Optimization steps on model reaction.


Entry	Catalyst	System	% Yield^a^
1	SiW_9_Ni_3_	Homogeneous	81
2	Zn_3_Al-LDH	Heterogeneous	62
3	SiW_9_Ni_3_@Zn_3_Al	Heterogeneous	94

Reaction condition: 1 mol% catalyst, 1 mmol of aminopyridine, 1 mmol of benzaldehyde, 1.2 mmol of cyclohexyl isocyanide, 35 °C, 1 h at solvent-free condition.

^a^Isolated yield.

To investigate the role of both SiW_9_Ni_3_ and Zn_3_Al–NO_3_ components in the progress of model reaction under optimized reaction conditions, a series of control experiments was performed (Table 1). It can be inferred from the results that the homogeneous form of SiW_9_Ni_3_ (Table 1, entry 1) exhibited a higher activity in comparison with the host Zn_3_Al-LDH (Table 1, entry 2) under identical conditions. The above results indicate that the inserted POM anions in the SiW_9_Ni_3_@Zn_3_Al composite are probably the main catalytic active sites. Due to the higher catalytic performance of SiW_9_Ni_3_@Zn_3_Al composite (Table 1, entry 3) than either of the individual constituents alone, it can be say that intercalating of SiW_9_Ni_3_ anions into the Zn_3_Al–NO_3_ interlayers can combine both Bronsted/Lewis acid sites of POM anions and Lewis acid effect of Zn^2+^ of Zn_3_Al–NO_3_ layered double hydroxide^37,49,50^. The cooperative effect of this catalytic system was demonstrated by the synthesis of aminoimidazopyridines with a good yield.

According to Table 2S, to expand the generality of this process, a series of substrates 1, 2 and 3 were examined with SiW_9_Ni_3_@Zn_3_Al as the catalyst under the optimized conditions. The results show that all the aldehyde derivatives of the isocyanides and aminopyridines offered good to excellent yields. A closely look to the data displayed in the Table 2S, it is observed that Aryl aldehydes with electron-deficient groups (entries 2, 3, 4) accelerate the reaction compared to benzene (entry 1) while the electron-donating groups (entries 6–12) bound to the 2-aminopyridine ring required longer reaction times albeit with lower yields (ESI).

In order to ascertain the heterogeneity of the SiW_9_Ni_3_@Zn_3_Al catalyst, hot filtration experiment was carried out. The catalyst was filtered from the reaction media after short reaction time. Then the reaction was checked if it proceeds in the absence of the catalyst under the same reaction conditions or not. However, it was observed that upon removal of catalyst no progress has been occurred even after carrying out the reaction mixture for longer duration confirming that the catalysis was true heterogeneous.

To investigate the recycling of the SiW_9_Ni_3_@Zn_3_Al, six reaction cycles have been tested. In each cycle, the solid catalyst was centrifuged and readily filtered from the reaction medium and then used in the next cycle. As illustrated in Fig. 1S, the catalyst shows high catalytic performance at least in 5 consecutive runs. However, the reaction time increased after fifth run (100 min). The FT-IR spectrum of the used catalyst revealed that the structure of the SiW_9_Ni_3_@Zn_3_Al catalyst almost retained its structural composition, even after six consecutive runs (Fig. 1Sb). Also, XRD spectra of the fresh and reused catalyst confirm the retention of SiW_9_Ni_3_@Zn_3_Al structural integrity (ESI).

## Conclusions

In summary, the POM anions of SiW_9_Ni_3_ has been confirmed to intercalated into the interlayers of Zn_3_Al-LDH, leading to the formation of the SiW_9_Ni_3_@Zn_3_Al via the selective ion-exchange synthetic approach. In the synthesis of amino imidazopyridines via three-component Ugi-like reaction (isocyanide-based), SiW_9_Ni_3_@Zn_3_Al illustrates better catalytic performance (yield: ~ 98%) than those of SiW_9_Ni_3_ and Zn_3_Al–NO_3_, both individual constituents. Moreover, the SiW_9_Ni_3_@Zn_3_Al composite showed remarkable catalytic activity for the synthesis of amino imidazothiazole with high yields under mild and S-F conditions as well. In particular, Zn_3_Al–NO_3_ not only shows excellent capacity to act as a support for highly dispersed and firmly immobilized SiW_9_Ni_3_ guests but also contributes to improve the catalytic activity of composite via the synergic effect of Lewis acid effect of Zn^2+^ of Zn_3_Al–NO_3_ layered double hydroxide and Bronsted/Lewis acid sites of POM anions. Additionally, according to SEM and BET results, the separation of the LDH layers by the uniform dispersion of POM entities in the LDH gallery and significant increase in specific surface area is not negligible in the catalytic performance of SiW_9_Ni_3_@Zn_3_Al. Furthermore, recovery experiments exhibit that the catalyst can be easily separated from the reaction media and be reused for more than 5 cycles without obvious decrease in catalytic performance.

## Supplementary Information

Supplementary Information.

## Data Availability

All data generated or analysed during this study are included in this published article (and its Supplementary Information files).
