# Supplementary material for: Nickel substituted polyoxometalates in layered double hydroxides as metal-based nanomaterial of POM–LDH for green catalysis effects
Source: Sci Rep. 2023 Mar 13;13:4114. doi: 10.1038/s41598-023-31356-7 (PMC10011597; doi:10.1038/s41598-023-31356-7)
Supplement: Supplementary file 1 — Supplementary Information. [file 41598_2023_31356_MOESM1_ESM.pdf]

Electronic Supporting Information

Nickel Substituted Polyoxometalates in Layered Double Hydroxides as Metal-based Nanomaterial of POM-LDH for Green Catalysis Effects

Azra Ghiasi Moaser, [a] Ahmad Gholami Afkham, [b] Roushan Khoshnavazi, *[b] Sadegh Rostamnia, *[a]

[a] *Organic and Nano Group (ONG), Department of Chemistry, Iran University of Science and Technology (IUST), PO Box 16846-13114, Tehran, Iran. Email: rostamnia@iust.ac.ir;srostamnia@gmail.com*

[b] *Department of Chemistry, University of Kurdistan P.O. Box 66135-416, Sanandaj, Iran. Email: r.khoshnavazi@uok.ac.ir*

Experimental

Materials and apparatus

All the chemicals were purchased from commercial companies and used without further purification. Powder X-ray diffraction (XRD) patterns were recorded on a Philips X'Pert MPD diffractometer equipped with Cu Kα radiation (λ=1.54056 Å) and operated at 40 kV 30 mA. FT-IR spectra were recorded on a Bruker model vector 22 Fourier transform spectrophotometer, using KBr Pellet. Surface areas and pore size distributions were investigated using nitrogen physisorption at 77 K on a Micromeritics Tristar II Plus surface area analyzer. The Scanning electron microscopy (SEM) images and energy dispersive X-ray (EDX) analytical data were determined using FESEM-TESCAN MIRA3 scanning electron microscope equipped with an EDX detector. Transmission electron microscopy (TEM) was performed with a Zeiss-EM10C microscope operating at 80 kV. Thermogravimetric analyses (TGA) were carried out by a STA PT-1000 LINSEIS apparatus.

Preparation of SiW_9Ni_3@Zn_3Al nanocomposite

The preparation of SiW_9Ni_3@Zn_3Al nanocomposite was performed through a three-step procedure: 1) synthesis of α-$[SiW_9O_{37}\{Ni(H_2O)\}_3]^{-10}$, 2) hydrothermal synthesis of the Zn_3Al-NO_3 layered double hydroxide and finally 3) intercalation of the $[SiW_9O_{37}\{Ni(H_2O)\}_3]^{-10}$ anions into the Zn_3Al-NO_3 via anion exchange process under N_2 atmosphere. Decarbonated-deionized water is used in experiments. It is prepared by boiling and bubbling nitrogen gas into the deionized water to remove the dissolved CO_2.

Synthesis of α-$Na_{10}[SiW_9O_{34}].18H_2O$ (SiW_9):

Firstly, 91 g of Sodium tungstate was dissolved in 100 mL of water. After clarifying, 5.5 g of sodium silicate was added to a vigorously stirred solution. Then 65 mL of HCl acid (6 M) was added with stirring. In the next step, the solution was boiled to concentrate it to half of its volume. After cooling down, the solution was filtered, then 20 g of anhydrous sodium carbonate was added to the filtrate. Then, the solution was gently stirred for 20 minutes. Finally, the sodium salt of the a-9-tungstosilicate precipitated.

Synthesis of α-$[SiW_9O_{37}\{Ni(H_2O)\}_3]^{-10}$ (SiW_9Ni_3):

Firstly, 3.4 g (12 mmol) $NiSO_4.7H_2O$ was dissolved in 150 mL of sodium acetate (0.5 M). In the next step, 11.2 g (4mmol) SiW_9 was added to the solution at 70 ºC. After cooling to room temperature, a solution of 4.2 g KCl in 12 mL water was added to yield an iridescent green precipitate. The green product was recrystallized from hot water.

Synthesis of Zn_3Al-NO_3 (Zn_3Al-LDH):

In a typical experiment, a solution of 7.8 g $Zn(NO_3)_2{\cdot}4H_2O$ (0.03 mol) and 3.8 g $Al(NO_3)_3{\cdot}9H_2O$ (0.01 mol) in 100 ml decarbonated H_2O was mixed with a solution of 3.2 g NaOH (0.08 mol) in 100 ml of decarbonated H_2O. In two minutes, the resulting slurry was transferred to autoclave, aged for 12 hours at 100 ºC. After cooling to room temperature, it was washed with boiling decarbonated H_2O for three times.

Preparation of SiW_9Ni_3@Zn_3Al

In a typical procedure, a solution of SiW_9Ni_3 (3.2 g, 1.14 mmol) in 40 mL of decarbonated water was added dropwise to the slurry of Zn_3Al-NO_3 under N_2 atmosphere while vigorously stirring and then stirred for 5 hours at 60 ℃. Finally, the green precipitate of SiW_9Ni_3@Zn_3Al was filtered, washed with boiling decarbonated water for three times and dried at 60 ℃ overnight under vacuum.

General procedure for S-F preparation of the aminoimidazoles

In a typical procedure, to a mixture of 2-aminopyridines (1 mmol) and aldehyde (1 mmol) the SiW_9Ni_3@Zn_3Al catalyst (1 mol%) was added under solvent-free condition at room temperature. The resulting reaction mixture was then allowed to stir for 5 min. Afterwards, alkyl isocyanide (1.2 mmol) was added to the reaction mixture and stirred at 35 ℃ for appropriate time. Progress of the reaction was checked by TLC. At the end of the reaction, after cooling the reaction mixture, CH_2Cl_2 (4 mL) was added. The catalyst was easily separated (after the adding of CH_2Cl_2) from the product by centrifuged (3000 rpm for 10 minutes). Afterwards, it was washed with dichloromethane and diethyl ether to remove residual product and dried under vacuum to reuse in the next run. The corresponding product was separated from the reaction medium by extraction. The all of the isolated products gave satisfactory spectral data and physical data [(^{1}H NMR and ^{13}C NMR)] compared with those reported in our previous reports[1-2].

Table 1S. Optimization steps on model reaction. [a]

Entry	Solvent	Mol% Cat.	Temp. (°C)	Time (h)	% Yield [b]
1	H_2O	0.5	r.t	0.5	17
2	H_2O	0.5	50	0.5	32
3	Toluene	0.5	r.t	1	20
4	EtOH	0.5	r.t	0.5	32
5	EtOH	0.5	30	0.5	39
6	MeOH	0.5	r.t	0.5	15
7	CH_2Cl_2	0.5	r.t	0.5	20
8	S-F	0.5	r.t	0.5	38
9	S-F	0.5	35	0.5	65
10	S-F	0.5	r.t	1	60
11	S-F	0.5	35	1	93
12	S-F	0.5	35	0.5	59
13	S-F	0.5	35	1	67
14	S-F	1	r.t	0.5	41
15	S-F	1	r.t	1	55
**16**	**S-F**	**1**	**35**	**1**	**94**
17	S-F	1	45	1	88
18	S-F	2	r.t	1	49
19	S-F	2	35	1	94
20	S-F	2	50	1	94
21	S-F	3	r.t	1	68
22	S-F	5	r.t	1	71

[a] Reaction condition: aminopyridine (1 mmol), benzaldehyde (1 mmol), cyclohexyl isocyanide (1.2 mmol), solvent (3 mL). [b] Isolated yield.

Table 2S. One-pot synthesis of imidazopyridines using $SiW_9Ni_3@Zn_3Al$ as the catalyst. [a]

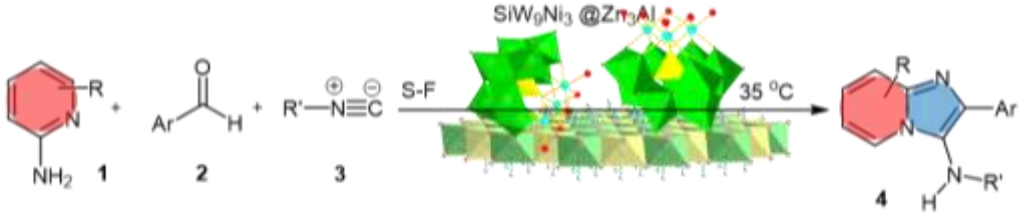

Entry	R	Ar	R'	Time (min)	% Yield [c]
1	H	C_6H_5	Cycl-Hex [b]	60	96
2	H	4-NO_2-C_6H_4	Cycl-Hex	45	96
3	H	4-Cl-C_6H_4	Cycl-Hex	45	94
4	H	3-NO_2-C_6H_4	Cycl-Hex	45	95
5	H	C_6H_5	^{t}Bu-	60	86

6	6-CH_3	C_6H_5	Cycl-Hex	70	84
7	6-CH_3	4-Cl-C_6H_4	Cycl-Hex	60	93
8	6-CH_3	3-NO_2-C_6H_4	Cycl-Hex	60	92
9	6-CH_3	4-NO_2-C_6H_4	Cycl-Hex	60	87
10	6-CH_3	4-NO_2-C_6H_4	^{t}Bu-	60	89
11	5-CH_3	C_6H_5	Cycl-Hex	70	90
12	5-CH_3	4-Cl-C_6H_4	Cycl-Hex	60	89

[a] Reaction condition: aminopyridine (1 mmol), aldehyde (1 mmol), isocyanide (1.2 mmol). [b] Cycl-Hex (cyclohexyl).

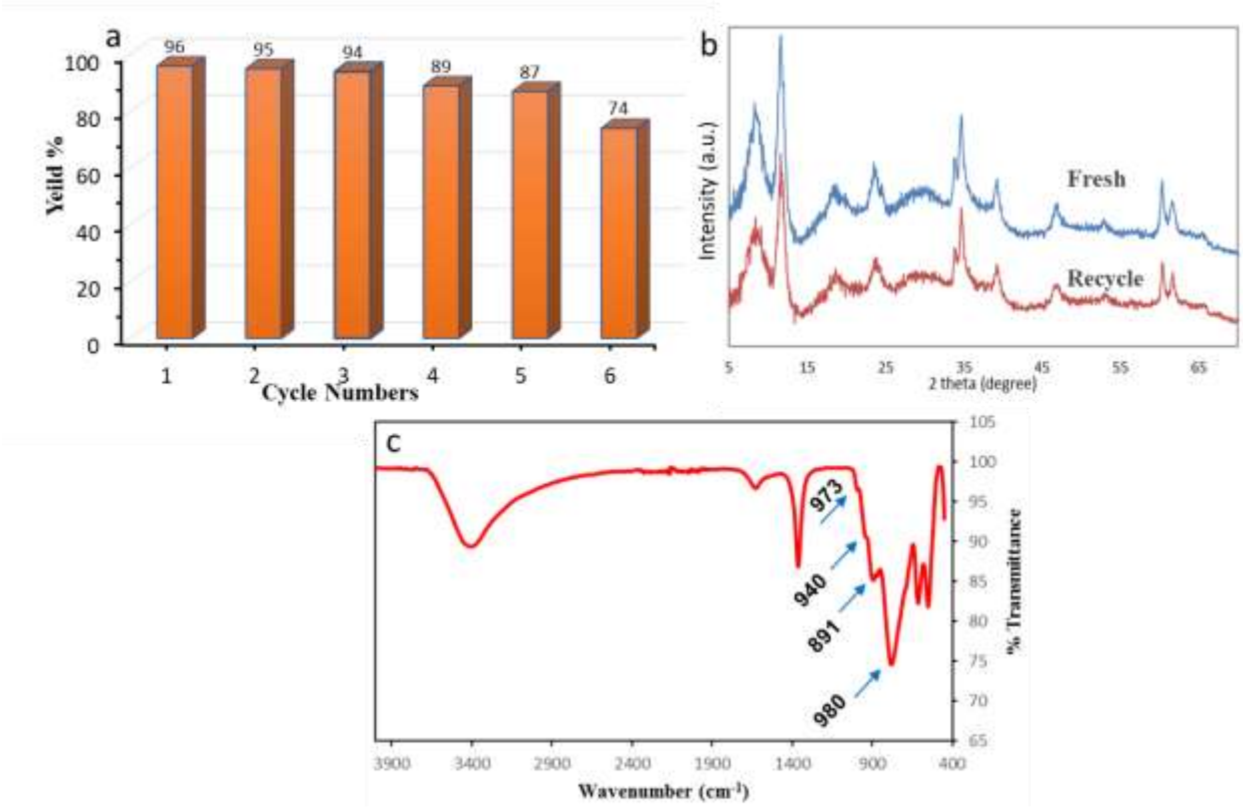

Figure 1S. (a) The reusability of $SiW_9Ni_3@Zn_3Al$ for the Ugi-like multicomponent synthesis of imidazopyridines (b) FT-IR spectrum of recycled $SiW_9Ni_3@Zn_3Al$ after six-time recycling.

References:

1) S. Rostamnia and M. Jafari, *Appl. Organomet. Chem*., **2017**, 31, 1–6.

2) S. Rostamnia, K. Lamei, M. Mohammadquli, M. Sheykhan and A. Heydari, *Tetrahedron Lett*., **2012**, 53, 5257–5260.
